# Cathelicidin CATH-2 suppresses the NF-κB/ROS/NLRP3 signaling pathway via regulating mTOR-dependent autophagy during *Streptococcus suis* infection

**DOI:** 10.1186/s13567-025-01694-7

**Published:** 2026-01-23

**Authors:** Liuyi Xu, Yilin Lu, Shichao Xu, Yuqian Liu, Hongdou Liu, Tingting Zhang, Yandi Pan, Yi Lu, Zhouyuan Wang, Xuefeng Cao, Zhiwei Li, Rendong Fang, Lianci Peng

**Affiliations:** 1 Joint International Research Laboratory of Animal Health and Animal Food Safety, College of Veterinary Medicine, No.2 Tiansheng Road, Beibei District, Chongqing, 400715 China; 2 National Center of Technology Innovation for Pigs, Chongqing, China; 3 Kunming Hemeihua Feed Limited Company, Kunming, China

**Keywords:** Cathelicidin-2, *Streptococcus suis*, anti-inflammatory response, autophagy

## Abstract

Cathelicidin CATH-2 has been reported to exert potent anti-inflammatory activity in different species though neutralizing stimuli such as lipopolysaccharide (LPS) and lipoteichoic acid (LTA). CATH-2 has been shown to inhibit *Streptococcus suis (S. suis)*-induced activation of dendritic cells and macrophages by binding to LTA. However, the exact mechanism of this prophylactically anti-inflammatory activity remains unclear. Therefore, we investigated the anti-inflammatory activity and mechanism of CATH-2 in mice peritoneal macrophages pretreated with CATH-2 followed by *S. suis* infection. The results showed that CATH-2 pretreatment significantly reduced *S. suis*-induced transcription and secretion of interleukin (IL)-1β, IL-6, and IL-12. CATH-2 also downregulated NOD-, LRR-, and pyrin domain-containing protein 3 (NLRP3) expression and apoptosis-associated speck-like protein containing a CARD (ASC) oligomerization, and inhibited the maturation of IL-1β, suggesting that CATH-2 inhibits NLRP3 activation. In addition, CATH-2 significantly inhibited *S. suis*-induced phosphorylation of p65 and extracellular signal-regulated kinase (ERK). Further study showed that CATH-2 inhibited *S. suis*-induced reactive oxygen species (ROS) by upregulating the expression of ROS scavenging genes including catalase (*CAT*) and superoxide dismutase 1 (*SOD1*). Mechanistically, transcriptome analysis revealed that CATH-2 regulated the protein kinase B (ATK)/mammalian target of rapamycin (mTOR) pathway, which was evident by the downregulation of phosphorylated (p)-ATK and p-mTOR induced by CATH-2. Notably, CATH-2 induced autophagy and autophagic flux. Inhibition of mTOR using rapamycin enhanced the CATH-2-induced autophagic efficacy, demonstrating that CATH-2 induces mTOR-dependent autophagy. However, inhibition of autophagy using 3-methyladenine (3-MA) reversed the reduction in the expression of p-p65, p-ERK, and IL-1β induced by CATH-2. Our study reveals that CATH-2 inhibits the nuclear factor kappa-B (NF-κB)/NLRP3-mediated inflammatory response through the induction of mTOR-dependent autophagy during *S. suis* infection, which provides new insight into the anti-inflammatory pathways of antimicrobial peptides.

**Supplementary Information:**

The online version contains supplementary material available at 10.1186/s13567-025-01694-7.

## Introduction

Antimicrobial peptides (AMPs) are naturally occurring peptides that are expressed in different species including invertebrates, plants, and microorganisms, being able to control microbial infections by direct antimicrobial activity or modulating the host immune response [1, 2]. So far, the antimicrobial peptide database has catalogued more than 3300 natural antimicrobial peptides [3]. Although these AMPs exhibit variation in sequence, they can be broadly classified into four structures including α-helical, β-sheet, cyclic, and extended flexible loop [4]. These varied sequences and structures of natural AMPs contribute to the design and development of novel therapeutic peptide analogs, which has great significance for addressing the global antibiotic resistance crisis [5, 6].

Cathelicidins, one of the families of AMPs, have multiple functions including antimicrobial and immunomodulatory activities [7, 8]. During pathogenic infection, cathelicidins are significantly upregulated and exert broad antimicrobial activity against bacteria [9], viruses [10, 11], fungi [12], and parasites [13]. Owing to their nonspecific targeting of microorganisms, cathelicidins depend on positive charge to interact with their membrane, which is less likely to develop drug resistance, leading to their consideration as promising alternatives or additives to antibiotics. Besides direct antimicrobial activity, cathelicidins attract further attention owing to their immunomodulatory functions, especially on anti-inflammatory activity, which have been extensively studied via neutralizing LPS or LTA to block Toll-like receptor (TLR)2 or TLR4 activation [14–16]. Cathelicidins also improve bacterial DNA uptake into macrophages to enhance TLR9 activation [17]. Our recent study reported that chicken CATH-2 as a second signal activates the NLRP3 inflammasome in LPS-primed murine macrophages and neutrophils [18, 19]. Furthermore, the antimicrobial efficacy of the administration of cathelicidins in vivo across species has also been identified [15, 20, 21]. CATH-2 pretreatment in chicken embryos by *in ovo* injection reduces avian pathogenic *Escherichia coli*-induced mortality and morbidity [22]. CATH-2 administration into the yolk of zebrafish embryos delays *Salmonella enterica*-induced zebrafish mortality [23]. A recent study reported that CATH-2 induces “trained immunity” in human macrophages to enhance the cell immune response [24]. These studies demonstrate that the immunomodulatory property of cathelicidins protects the host against microbial infection.

*Streptococcus suis* causes severe infection in humans and pigs, leading to sepsis and meningitis. The lack of an effective vaccine and the extensive use of antibiotics lead to the emergence of multiresistant *S. suis* strains [25]. Therefore, there is an urgent need to develop new strategies to control *S. suis* infection. Since *S. suis* induces an inflammation storm and causes severe tissue damage [26], cathelicidins with anti-inflammatory functions could be developed as anti-infective agents. A recent study reported that CATH-2 pretreatment via subcutaneous injection 24 h or 7 days before *S. suis* infection reduces the clinical symptoms in mice [27]. An in vitro study in murine bone marrow-derived macrophages (BMDMs) showed that CATH-2 reduces *S. suis*-induced inflammatory cytokines [27]. This study suggested that CATH-2 may exert a protective effect via an anti-inflammatory function against *S. suis* infection. However, the exact anti-inflammatory mechanism was not elucidated.

To develop CATH-2 as a potential antimicrobial agent, we investigated the mechanism by which CATH-2 inhibits *S. suis*-induced inflammatory signaling pathway activation. Our results show that CATH-2 inhibits activation of the *S. suis*-induced NF-κB/ROS/NLRP3 signaling pathway. Importantly, we reveal a novel function of CATH-2 that induces autophagy, while CATH-2 suppresses activation of the mentioned inflammatory signaling pathway via autophagy induction. Our study broadens knowledge on the anti-inflammatory function of AMPs.

## Materials and methods

### Animals

Wild-type (WT) C57BL/6 mice (*n* = 35) were purchased from Chongqing Lepitt Biotechnology Co., Ltd. All mice were maintained in specific pathogen-free (SPF) conditions before being used at 8–10 weeks of age. All animal experiments were approved by the Southwest University Ethics Committee, Chongqing, China (permission no. IACUC-20240119-042, October 2024).

### Peptides

CATH-2 (amino acid sequence: RFGRFLRKIRRFRPKVTITIQGSARF-NH2) [28] was synthesized by China Peptides (Shanghai, China) using fluorenylmethoxycarbonyl protecting group (Fmoc) chemistry and purified by reverse-phase high-performance liquid chromatography to purity > 95%.

### Bacterial strains and culture conditions

*S. suis* serotype 2 strain SC-19 provided by Professor Xiangru Wang (College of Veterinary Medicine, Huazhong Agricultural University) was grown in tryptic soy broth (TSB; Beijing Luqiao Technology Co., LTD., China) or tryptic soy agar (TSA; Beijing Luqiao Technology, LTD., China) supplemented with 10% fetal bovine serum (FBS; Zhejiang Tianxing Biotechnology, China) at 37 °C.

### Preparation of mouse primary peritoneal macrophages

To collect peritoneal macrophages, mice were intraperitoneally injected with 4% thioglycolate for 3 or 4 days. Then, peritoneal lavage was collected to harvest cells, and cells were suspended in Roswell Park Memorial Institute (RPMI) 1640 with 10% FBS. After centrifugation, washing, and resuspension, cells were seeded in 48-well, 24-well, 12-well, or 100-mm cell culture plates at density of 2 × 10^5^ cells/well, 5 × 10^5^ cells/well, 1 × 10^6^ cells/well, or 6 × 10^6^ cells/plate, respectively, and incubated at 37 ℃ with 5% CO_2_ for at least 2 h to facilitate cell adherence. Nonadherent cells were removed. Adherent cells were washed before being used for the experiments as described below.

### *S. suis* infection of macrophages

Cells were first pretreated with CATH-2 (1.25 µM or 2.5 µM) for 6 h, and then peptides were washed away, followed by *S. suis* infection at a multiplicity of infection (MOI) of 5 for 2, 4, or 6 h. Next, gentamicin (250 μg/mL) was used to kill extracellular bacteria, and these cells were continued to incubation for the indicated times. To promote or inhibit autophagy, rapamycin (RAPA, 100 nM) and 3-methyladenine (3-MA, 5 mM) were added for 1 h prior to CATH-2 treatment. To inhibit reactive oxygen species (ROS), *N*-acetylcysteine (NAC, 2 mM) was added for 30 min prior to CATH-2 treatment.

### Cell viability

Cells were prepared in 48-well plates and treated with CATH-2 (1.25 µM and 2.5 µM) for 6 h. Then, cells were washed away and continued in incubation for an additional 24 h. Next, cell medium was replaced with 10% WST reagent (Biomed, Beijing, China) for 2 h. Finally, absorbance was measured at 450 nm with a microplate reader (Bio-Rad, Japan).

### Enzyme-linked immunosorbent assay (ELISA)

Cells were prepared in 48-well plates and treated with CATH-2 (1.25 µM and 2.5 µM) followed by bacterial infection as described above. After 24 h infection, supernatants were collected to determine the concentration of cytokines including IL-1β, IL-6, and IL-12 (Invitrogen, CA, USA) according to the manufacturer’s instructions.

### Quantitative real-time polymerase chain reaction (RT‑PCR)

Cells were prepared in 12-well plates and treated with CATH-2 (1.25 μM and 2.5 μM) followed by bacterial infection. After 3 and 6 h infection, total RNA was extracted by TRIzol reagent (Life Technologies Carlsbad, CA, USA) according to the manufacturer’s instructions. Then, complementary DNA (cDNA, 500 ng) was synthesized using PrimeScript^®^ RT reagent Kit (Takara, Japan). Finally, RT-PCR was performed using a CFX96 (Bio-Rad, USA). Primers used are presented in Table 1. Relative gene expression level was normalized against the expression level of β-actin.

**Table 1:** **RT-PCR oligonucleotide primers**

Primer	Forward (5′–3′)	Reverse (5′–3′)
IL-1β	GAAATGCCACCTTTTGACAGTG	TGGATGCTCTCATCAGGACAG
IL-6	CTGCAAGAGACTTCCATCCAG	AGTGGTATAGACAGGTCTGTTGG
IL-12	GTCCTCAGAAGCTAACCATCTCC	CCAGAGCCTATGACTCCATGTC
CAT	AGGCTCAGCTGACACAGTTC	ATGGAGAGACTCGGGACGAA
SOD1 AKT mTOR	GGAACCATCCACTTCGAGCA TGCACAAACGAGGGGAA CTGATCCTCAACGAGCTAGTTC	CCCATGCTGGCCTTCAGTTA CGCTGATCCACATCCTGA GGTCTTTGCAGTACTTGTCATG
Pdgfb	GAGTGTGGGCAGGGTTATTT	GAATCAGGCATCGAGACAGAC
Itga6	CATCACGGCTTCTGTGGAGATC	CATTGTCGTCTCCACATCCTTCC
Efna5	ATGTTGACGCTGCTCTTTCTGGT	CGTTGTCTGGGATTGCAGAGGA
F2r	TGAACCCCCGCTCATTCTTTC	CCAGCAGGACGCTTTCATTTTT
β-Actin	TGGAATCCTGTGGCATCCATGAAAC	TAAAACGCAGCTCAGTAACAGTCCG

### Western blotting analysis

Cells were prepared in 12-well plates and treated with CATH-2 (2.5 μM) followed by bacterial infection. After 1, 2, 4, or 24 h infection, supernatants were collected and concentrated using 20% (w/v) trichloroacetate, and cell lysates were collected using 1× sodium dodecyl sulfate (SDS) loading buffer (Beyotime, China). Subsequently, concentrated supernatants and cell lysates were subjected to 12% SDS polyacrylamide gel electrophoresis (PAGE) and then transferred onto a polyvinylidene difluoride (PVDF) membrane by electroblotting. Next, the membranes were blocked with 5% nonfat dry milk and then immunoblotted with the indicated primary antibodies (Abs) including anti-caspase-1 (AdipoGen, USA), anti-IL-1β (Bioss, China), anti-NLRP3 (Cell Signaling Technology, USA), anti-p65 (Beyotime, China), anti-phospho-p65 (p-p65; Proteintech, China), anti-ERK (Bioss, China), anti-phospho-ERK (p-ERK; Cell Signaling Technology, USA), anti-ASC (Santa Cruz Biotechnology, Inc), anti-AKT (Abcam, UK), anti-phospho-AKT (p-AKT; Abcam, UK), anti-mTOR (Abcam, UK), anti-phospho-mTOR (p-mTOR; Proteintech, China), anti-LC3 (Cell Signaling Technology, USA), anti-p62 (Proteintech, China), and anti-β-actin (Beyotime, China). Next day, the blots were incubated with horseradish peroxidase-conjugated goat anti-mouse/rabbit immunoglobulin G (IgG). Finally, the distinct protein bands were detected by enhanced chemiluminescence (ECL) detection reagent (Biosharp, China).

### ASC oligomerization

Cells were prepared in 12-well plates and treated with CATH-2 (2.5 µM) followed by bacterial infection. After 24 h infection, cells were lysed with cold phosphate-buffered saline (PBS) containing 0.5% Triton X-100, and then centrifuged at 13,000 rpm for 15 min at 4 ℃ to obtain cell pellets. Next, the pellets were washed twice with cold PBS and resuspended in 200 μL PBS. Subsequently, the resuspended pellets were cross-linked with 2 mM fresh disuccinimidyl suberate (DSS) at 37 ℃ for 30 min and then centrifuged at 13,000 rpm for 15 min at 4 ℃. Finally, the cross-linked pellets were dissolved in 30 μL 1× SDS-PAGE sample loading buffer and boiled for 5 min before western blotting analysis.

### In situ detection of ROS

Cells were prepared in 48-well plates and treated with CATH-2 (1.25 μM and 2.5 μM) followed by bacterial infection. After 6 h infection, cell medium was replaced by 5 μM dihydroethidium (DHE; Beyotime, China), and these cells were continued to incubation at 37 °C for 30 min. ROS production was observed under a fluorescence microscope (Olympus, Tokyo, Japan). Fluorescence intensity was analyzed using a fluorescence spectrophotometer (Biotek, USA).

### Transcriptome analysis

Cells were prepared in 100-mm cell culture plates (6 × 10^6^ cells/plate) and treated with CATH-2 (2.5 µM) followed by bacterial infection. After 3 h infection, cells were collected for RNA sequencing (RNA-seq) analysis in Shanghai Majorbio Bio-Pharm Technology Co., Ltd to identify differentially expressed genes (DEGs) in SC-19-infected cells with or without CATH-2 treatment.

Bioinformatic analysis of transcriptome data was performed on the Majorbio Cloud platform [29]. Functional annotation of all DGEs was performed using Gene Ontology (GO) [30] terms and Kyoto Encyclopedia of Genes and Genomes (KEGG) enrichment analysis [31]. Significant pathway activation is shown as *P* < 0.05.

### Analysis of autophagic flux

Cells were prepared in 24-well plates and infected with adenovirus expressing RFP-GFP-LC3B fusion protein (Ad-RFP-GFP-LC3B; HANBIO, Shanghai, China) at an MOI of 25 for 24 h. After viral infection, cells were washed and continued to incubation for 12 h. Then, cells were treated with CATH-2 (1.25 μM) for 6 h, followed by bacterial infection for 1 h. After treatment, cells were fixed with 4% paraformaldehyde (PFA) for 30 min. After fixation, cells were washed and permeabilized with 0.1% Triton X-100 in PBS for 10 min. Subsequently, 4′,6-diamidino-2-phenylindole (DAPI; Beyotime, Shanghai, China) was added for 5 min to visualize cell nuclei. Finally, cells were washed and maintained in antifade medium (Solarbio, Beijing, China). Cells were observed using fluorescence microscopy (Olympus, Tokyo, Japan).

### Statistical analysis

All data are presented as the mean ± standard error of the mean (SEM) of three independent experiments for each group (*n* = 3). One-way analysis of variance (ANOVA) was used to analyze statistical significance among different groups. All graphs were generated using GraphPad Prism software (San Diego, CA, USA). Statistical significance is shown as *P < 0.05, **P < 0.01, and ***P < 0.001, or “ns” for not significant.

## Results

### CATH-2 inhibits SC-19-induced transcription and secretion of inflammatory cytokines in macrophages

**Figure 1:**
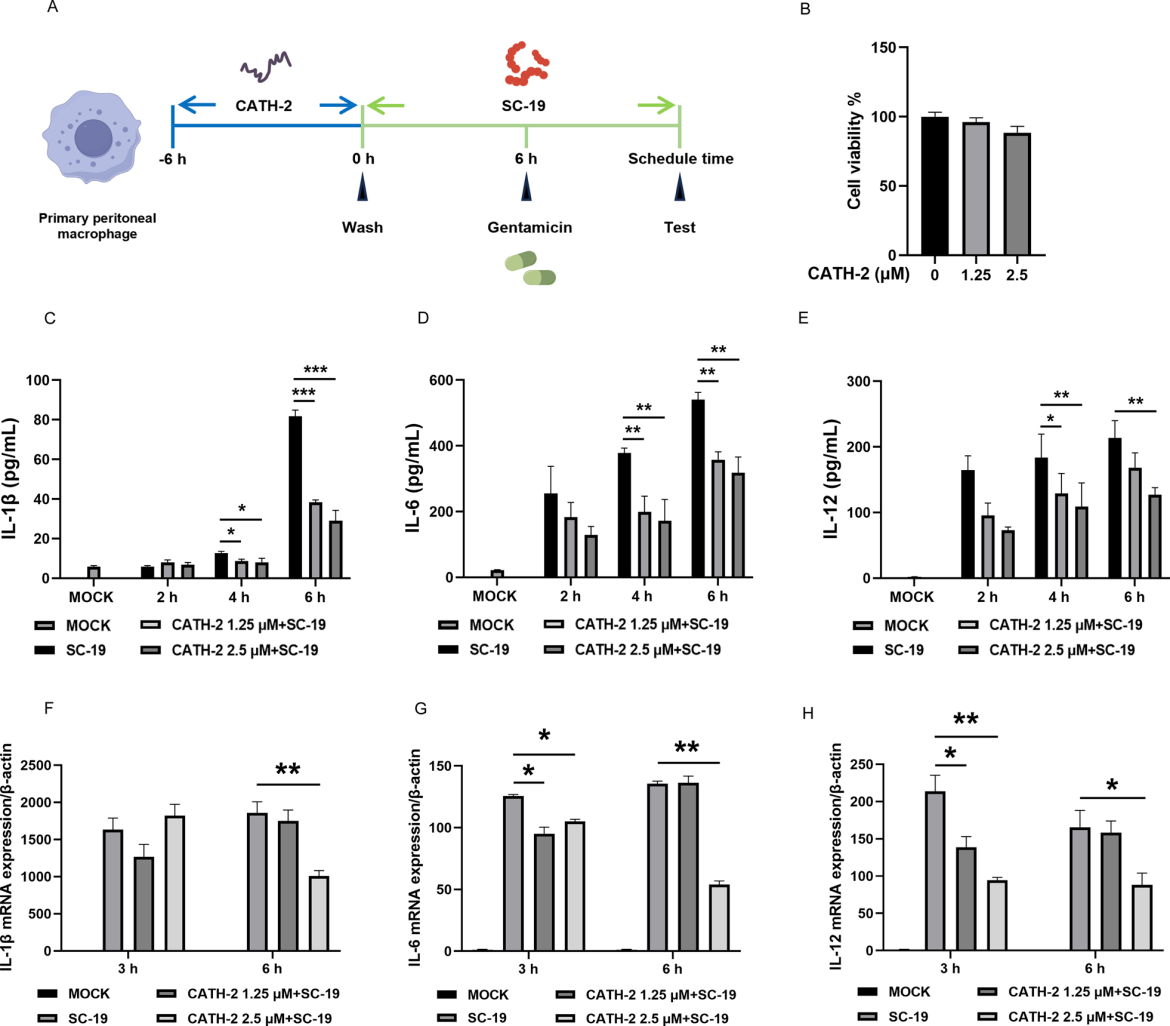
**CATH-2 inhibits SC-19-induced transcription and secretion of inflammatory cytokines in macrophages**. Cells were pretreated with CATH-2 (1.25 μM and 2.5 μM) for 6 h prior to SC-19 (MOI of 5) infection. After 2, 4, or 6 h of infection, gentamicin was added to cells for the indicated times. (**A**) Flowchart of the experimental setup. CATH-2 (1.25 μM and 2.5 μM) was added to cells for 6 h, and then washed away, followed by incubation for another 24 h. Cell viability was determined using the CCK-8 kit (**B**). After 24 h of infection, cell supernatants were collected. ELISA was performed to determine the level of inflammatory cytokines, including IL-1β (**C**), IL-6 (**D**), and IL-12 (**E**). After 3 and 6 h infection, RT-PCR was performed to determine the mRNA level of IL-1β (**F**), IL-6 (**G**), and IL-12 (**H**). Data presented as mean ± SEM of three independent experiments with triplicate samples per group. *P ≤ 0.05, **P ≤ 0.01, ***P ≤ 0.001.

To investigate the anti-inflammatory activity of CATH-2 in mice macrophages during *S. suis* infection, cells were pretreated with CATH-2 at sub-minimum inhibitory concentration (MIC; 1.25 µM and 2.5 µM) for 6 h, and then peptides were washed away, followed by bacterial infection as an experimental setup to avoid the direct effect of CATH-2 on bacterial killing or LTA neutralization (Figure 1A). The results showed that CATH-2 did not affect cell viability at 1.25 µM or 2.5 µM (Figure 1B). Next, to determine the optimal SC-19 infection time to induce a high level of inflammatory response, gentamicin was added to kill extracellular bacteria at 2, 4, and 6 h post-infection, followed by continued culture until 24 h. Then, supernatants were collected to determine the concentration of cytokines. The results showed that SC-19 induced production of IL-1β and IL-6 in a time-dependent manner during infection and also induced a high level of IL-12 production, whereas CATH-2 reduced such production of these inflammatory cytokines in a concentration-dependent manner (Figures 1C–E). Therefore, an initial 6 h infection followed by additional gentamicin was selected as the optimal experimental setup. In addition, the transcription levels of these inflammatory cytokines were detected using qPCR at 3 and 6 h post-infection. The results showed that SC-19 induced a high level of messenger RNA (mRNA) expression of IL-1β, IL-6, and IL-12 while CATH-2 decreased the mRNA expression of these cytokines (Figures 1F–H). These results indicate that CATH-2 effectively inhibits SC-19-induced transcription and secretion of inflammatory cytokines in macrophages.

### CATH-2 inhibits SC-19-induced NLRP3/MAPK/NF-κB signaling pathway activation in macrophages

**Figure 2:**
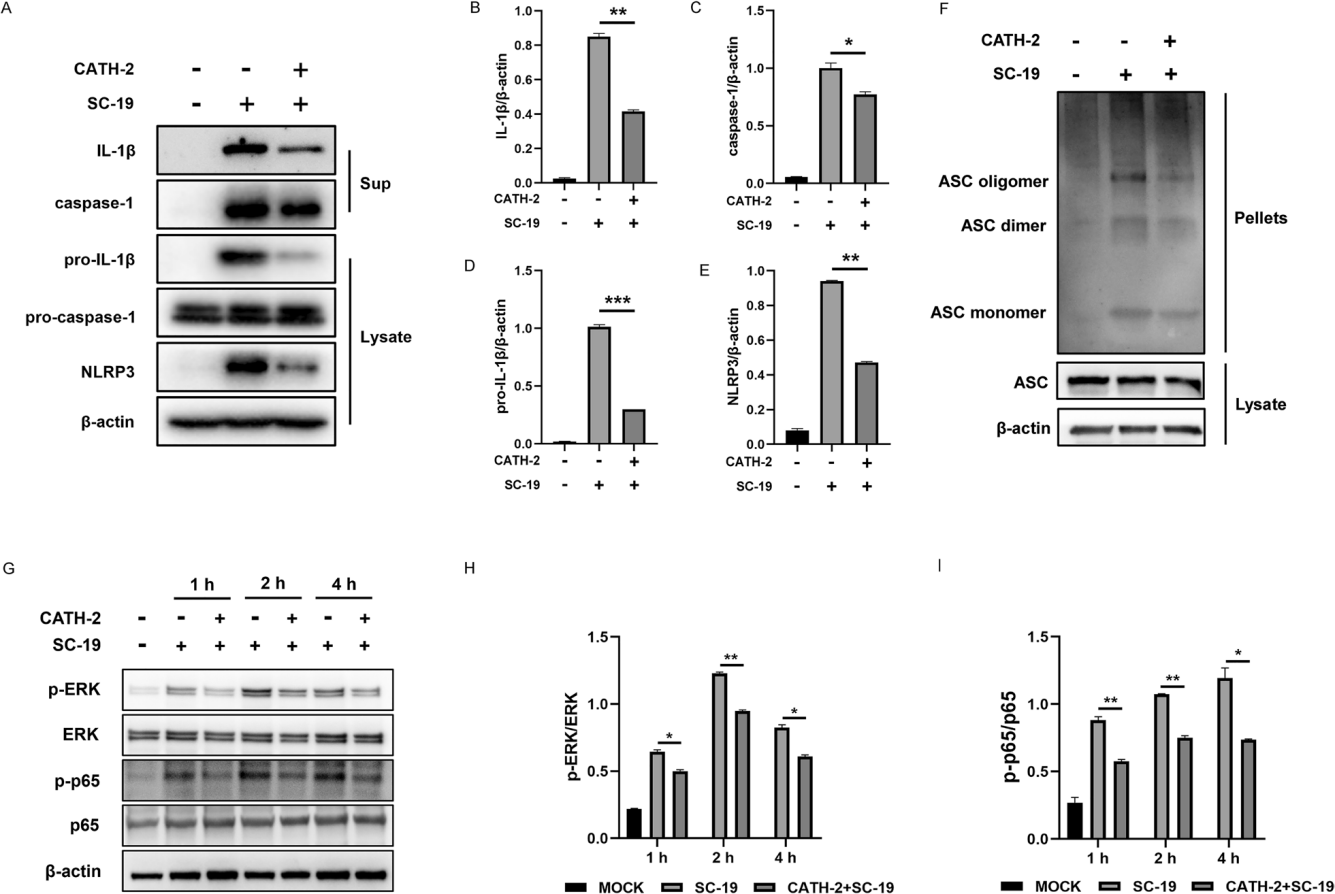
**CATH-2 inhibits SC-19-induced NLRP3/MAPK/NF-κB signaling pathway in macrophages.** Cells were pretreated with CATH-2 (2.5 μM) for 6 h and then infected with SC-19 at an MOI of 5 for 6 h. Then, gentamicin (250 μg/mL) was added to kill extracellular bacteria for the indicated times. After 24 h of infection, cell supernatants and lysates were collected. Western blotting was performed to detect the expression of proteins, including IL-1β, caspase-1, pro-IL-1β, pro-caspase-1, and NLRP3 (**A**). ImageJ was used to quantify the ratio of IL-1β (**B**), caspase-1 (**C**), pro-IL-1β (**D**), and NLRP3 (**E**) to β-actin. The level of ASC oligomerization was detected (**F**). After 1, 2, or 4 h of infection, cell lysates were collected to detect NF-κB and MAPK signaling pathway activation (**G**). ImageJ was used to quantify the p-ERK/ERK (**H**) and p-p65/p65 (**I**) ratios. Data presented as mean ± SEM of three independent experiments with triplicate samples per group. *P ≤ 0.05, **P ≤ 0.01, ***P ≤ 0.001.

It has been reported that *S. suis* triggers streptococcal toxic shock syndrome (STSLS) and cytokine storm via NLRP3 inflammasome activation. In addition, the transcription of these inflammatory cytokines depends on NF-κB activation, and these inflammatory responses are also regulated by other signaling pathways such as mitogen-activated protein kinase (MAPK) [26, 32]. Therefore, we investigated the effect of CATH-2 on the inflammatory signaling pathway including NLRP3, NF-κB, and MAPK during *S. suis* infection. The results showed that CATH-2 significantly downregulated SC-19-induced protein expression of pro-IL-1β and NLRP3 and reduced maturation of IL-1β and caspase-1, whereas it did not affect the expression of pro-caspase-1 (Figures 2A–E), suggesting that CATH-2 inhibits NLRP3 inflammasome activation. Full NLRP3 inflammasome activation is required to recruit caspase-1 via assembled ASC. Therefore, we detected the effect of CATH-2 on ASC expression. The results showed that CATH-2 downregulated SC-19-induced ASC oligomerization but did not affect the expression of total ASC (Figure 2F), indicating that CATH-2 inhibits NLRP3 inflammasome assembly. Furthermore, SC-19 induced phosphorylation of p65 and ERK at 1, 2, and 4 h post-infection, but CATH-2 significantly inhibited their phosphorylation (Figures 2G–I), demonstrating that CATH-2 inhibits MAPK/NF-κB pathway activation. These results suggest that CATH-2 suppresses SC-19-induced inflammatory response by the NLRP3/MAPK/NF-κB signaling pathway.

### CATH-2 inhibits SC-19-induced ROS in macrophages

**Figure 3:**
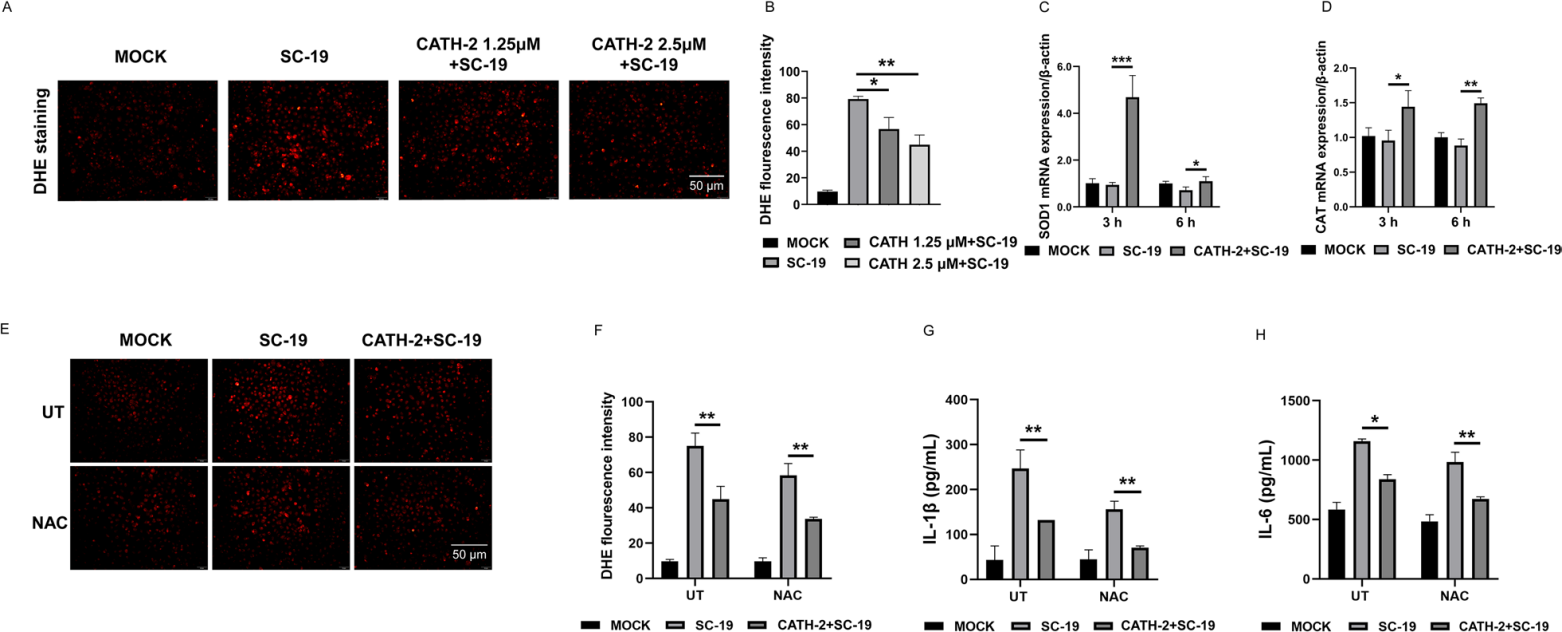
**CATH-2 inhibits SC-19-induced ROS in macrophages.** Cells were pretreated with CATH-2 (1.25 μM and 2.5 μM) for 6 h and then infected with SC-19 at an MOI of 5 for 6 h. Next, the DHE fluorescent probe was added for 30 min. Representative images of ROS (red) are shown by fluorescence staining (**A**). A fluorescence spectrophotometer was used to quantify the level of intracellular ROS (**B**). RT-PCR was used to determine the mRNA expression of *SOD1* (**C**) and *CAT* (**D**). Cells were incubated with NAC for 30 min prior to CATH-2 (2.5 μM) treatment followed by bacterial infection. Under treatment with NAC, DHE fluorescence images were taken (**E**). A fluorescence spectrophotometer was used to quantify the level of intracellular ROS (**F**). Cells were incubated with NAC for 30 min prior to CATH-2 (2.5 μM) treatment, followed by bacterial infection. After 24 h of infection, cell supernatants were collected. ELISA was performed to determine the level of IL-1β (**G**) and IL-6 (**H**). Data presented as mean ± SEM of three independent experiments with triplicate samples per experiment. *P ≤ 0.05, **P ≤ 0.01, ***P ≤ 0.001.

ROS production is considered to be a common signal of NLRP3 inflammasome activation [33]. Therefore, we investigated the effect of CATH-2 on ROS during *S. suis* infection via DHE. The results showed that SC-19 induced ROS production with a high level of fluorescence intensity of DHE, and CATH-2 decreased SC-19-induced fluorescence intensity of DHE (Figure 3A). Quantification of the DHE fluorescence intensity was carried out using a fluorescence spectrophotometer, which showed that CATH-2 reduced SC-19-induced ROS (Figure 3B). Next, the transcript levels of ROS scavenging genes including catalase (*CAT*) and superoxide dismutase 1 (*SOD1*) were determined by RT-PCR. The results showed that CATH-2 significantly enhanced mRNA expression of *CAT* and *SOD1*, which play important roles in the antioxidative response (Figures 3C, D), suggesting that CATH-2 eliminates ROS production during *S. suis* infection. To further investigate the role of ROS in the anti-inflammatory effect of CATH-2, the antioxidant acetylcysteine (NAC) was used to inhibit ROS production. The results showed that NAC reduced SC-19-induced ROS production and enhanced the inhibitory effect of CATH-2 on SC-19-induced ROS production (Figures 3E, F). Similarly, NAC significantly attenuated SC-19-induced secretion of IL-1β and IL-6 (Figures 3G, H), demonstrating that ROS mediate the inflammatory response during *S. suis* infection. Consistent with inhibitory effect of inflammatory response, NAC promoted the inhibitory effect of CATH-2 on SC-19-induced secretion of IL-1β and IL-6 (Figures 3G, H). These results suggest that CATH-2 suppresses the ROS-mediated inflammatory response during *S. suis* infection in macrophages.

### CATH-2 regulates the AKT/mTOR signaling pathway during *S. suis* infection from transcriptome analysis

**Figure 4:**
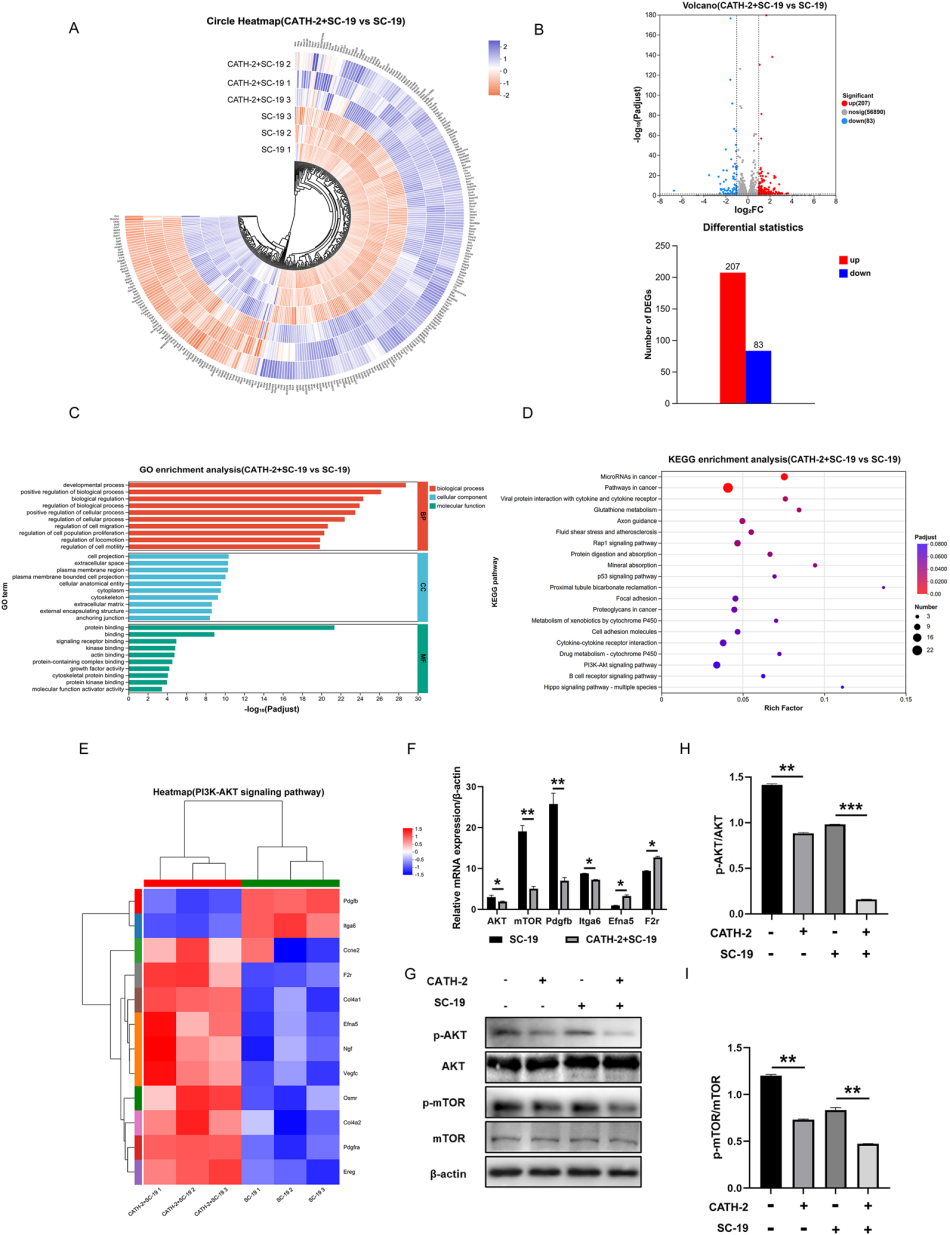
**CATH-2 regulates the AKT/mTOR signaling pathway during *S. suis*** **infection from transcriptome analysis**. Cells were pretreated with CATH-2 (2.5 μM) for 6 h and then infected with SC-19 at an MOI of 5 for 3 h. After infection, cells were collected for transcriptome sequencing. Clustering circle heatmap analysis of SC-19-infected cells treated with or without CATH-2 (**A**). Volcano plot and histogram of differential gene expressions (DGEs) in the transcriptome (**B**). The *X*-axis represents −log_10_(*P*_adjust_), while the *Y*-axis represents enriched terms. The top 10 significantly enriched GO terms of DGEs in biological process, cellular component, and molecular function categories (**C**). The *X*-axis represents the rich factor, and the *Y*-axis represents enriched terms, while bubble size and color represent gene count and *P*-value, respectively (**D**). Clustering heatmap analysis of DGEs in the PI3K/AKT signaling pathway (**E**). Cells were pretreated with CATH-2 (2.5 μM) for 6 h and then infected with SC-19 at an MOI of 5 for 1 h. RT-PCR was used to detect the transcription level of the genes of AKT, mTOR, Pdgfb, Itga6, Efna5, and F2r in the PI3K/AKT/mTOR signaling pathway (**F**). Cells were pretreated with CATH-2 (1.25 μM) for 6 h and then infected with SC-19 at an MOI of 5 for 1 h. Western blot was used to detect the expression of proteins, including p-AKT, AKT, p-mTOR, and mTOR (**G**). ImageJ was used to quantify the p-AKT/AKT (**H**) and p-mTOR/mTOR (**I**) ratios. *A*–*E* are representative of one independent experiment with triplicate samples per group. Data in *F*–*I* are presented as the mean ± SEM of three independent experiments with triplicate samples per group. *P ≤ 0.05, **P ≤ 0.01, ***P ≤ 0.001.

To investigate the exact inflammatory signaling pathway that CATH-2 regulates, we performed RNA-seq in CATH-2-treated macrophages followed by *S. suis* infection. The gene expression pattern is shown in the circle heatmap in Figure 4A, revealing DEGs in CATH-2 + SC-19-treated cells compared with SC-19-infected cells. RNA-seq analysis showed that CATH-2 significantly upregulated 207 genes and downregulated 83 genes during SC-19 infection, as shown in the volcano plot in Figure 4B. GO enrichment analysis showed that CATH-2-mediated DEGs were enriched in terms such as biological regulation, cellular process regulation, cell migration, signaling receptor binding, kinase binding, protein kinase binding, etc. (Figure 4C). KEGG enrichment analysis showed that CATH-2-mediated DEGs were enriched in different signaling pathways such as the Rap1 signaling pathway, protein digestion and absorption, p53 signaling pathway, cell adhesion molecules, cytokine–cytokine receptor interactions, and the phosphoinositide 3-kinase (PI3K)/AKT signaling pathway (Figure 4D). GO and KEGG enrichment analysis indicated that CATH-2 modulates cellular signaling pathways. Next, we further analyzed the CATH-2-mediated PI3K/AKT signaling pathway. Heatmap analysis revealed CATH-2-mediated DEGs enriched in the PI3K/AKT/mTOR signaling pathway (Figure 4E). To verify the CATH-2-mediated PI3K/AKT/mTOR signaling pathway during SC-19 infection, we determined the expression of the key genes and proteins. The results showed that CATH-2 significantly downregulated mRNA expression of AKT, mTOR, Pdgfb, and Itga6 but upregulated mRNA expression of Efna5 and F2r, which is upstream to mediate PI3K/AKT/mTOR (Figure 4F). Consistent with the mRNA expression, CATH-2 significantly reduced the phosphorylation of p-AKT and p-mTOR during SC-19 infection (Figures 4G–I). These results suggest that CATH-2 might regulate the PI3K/AKT/mTOR axis-mediated inflammatory response during *S. suis* infection.

### CATH-2 inhibits the SC-19-induced inflammatory response through mTOR-mediated autophagy in macrophages

**Figure 5:**
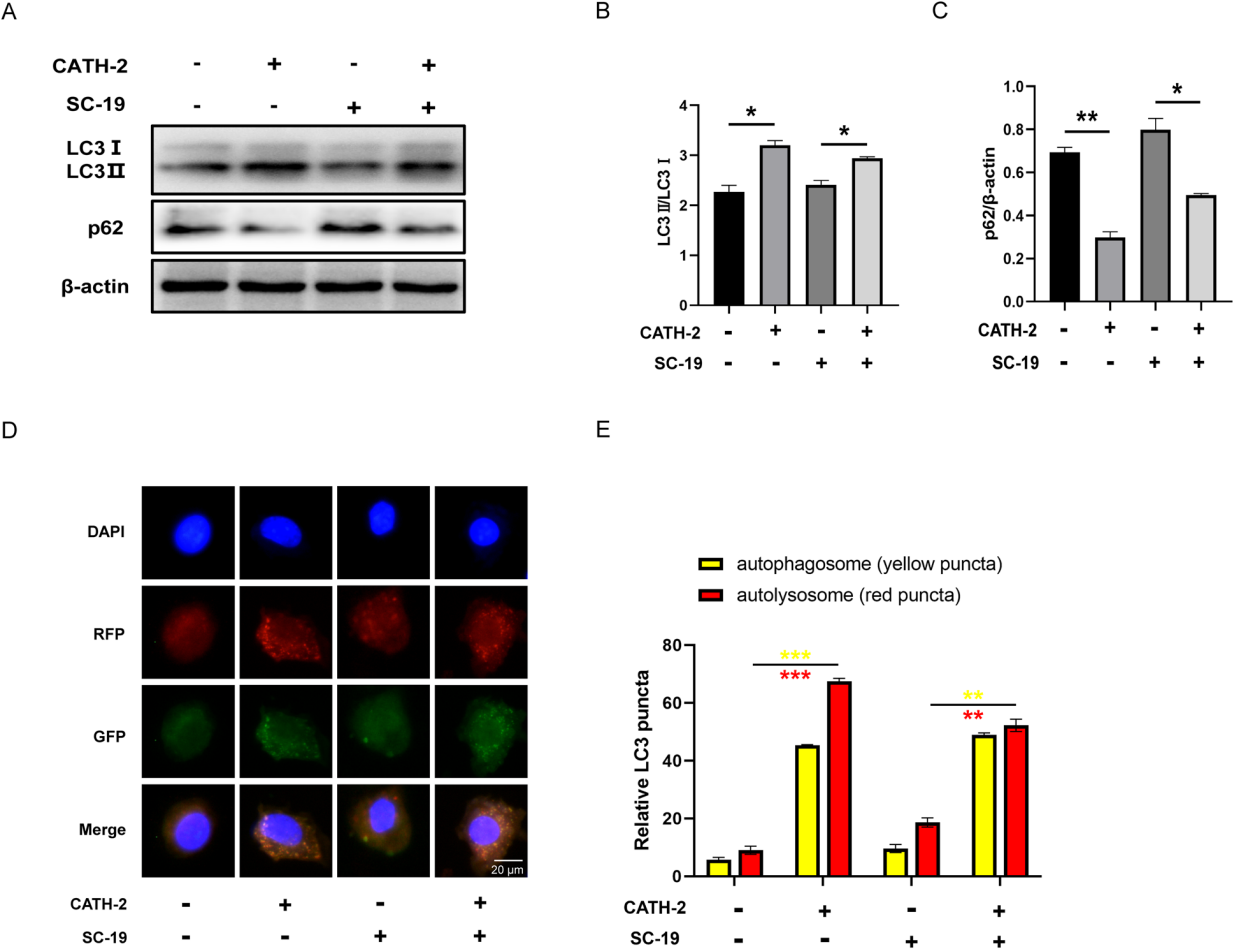
**CATH-2 induces autophagy and autophagic flux in macrophages.** Cells were pretreated with CATH-2 (1.25 μM) for 6 h and then infected with SC-19 at an MOI of 5 for 1 h. Western blotting was performed to determine the expression of proteins, including LC3 and p62 (**A**). ImageJ was used to quantify the LC3-II/LC3-I (**B**) and p62/β-actin (**C**) ratios. Cells were infected with adenovirus expressing RFP-GFP-LC3 at an MOI of 25, then cells were pretreated with CATH-2 (1.25 μM) for 6 h followed by SC-19 infection at an MOI of 5 for 1 h. Representative images of RFP-LC3 (red), GFP-LC3 (green), and DAPI (blue) under immunofluorescence staining (**D**). Quantification of yellow and red puncta (**E**). Data presented as mean ± SEM of three independent experiments with triplicate samples per group. *P ≤ 0.05, **P ≤ 0.01.

Since we have shown that CATH-2 mediated the PI3K/AKT/mTOR pathway, mTOR is generally regarded as a central regulator of autophagy [34]. Therefore, we investigated the effect of CATH-2 on autophagy during SC-19 infection. The results showed that CATH-2 alone and CATH-2 with SC-19 infection significantly increased the expression of LC3 protein, which is specifically recruited to autophagosome membranes (Figures 5A, B). SQSTM1 (known as p62) is responsible for the recruitment of specific targets to the phagosomes following degradation in the autolysosome together with polyubiquitinated target proteins. Our results showed that CATH-2 alone and CATH-2 with SC-19 infection significantly decreased the expression of p62 protein (Figures 5A, C). These results indicate that CATH-2 induces autophagy. Since autophagy is considered a dynamic process referred to as autophagic flux, we further investigated the change of autophagic flux via loading Ad-RFP-GFP-LC3B into cells. In the initial stage, autophagosomes were double-labeled with GPF (green) and RFP (red), showing yellow fluorescence. In the late stage, fusion of autophagosomes with lysosomes induces acidity, which leads to degradation of GFP fluorescence and consequently leaves more RFP fluorescence. Our results showed that CATH-2 alone increased the number of GFP-LC3 puncta (green) and RFP-LC3 puncta (red) as well as the number of yellow puncta (Figures 5D, E). However, SC-19 infection showed a decreased number of yellow and red puncta compared with CATH-2 treatment (Figures 5D, E), demonstrating that CATH-2 promotes the fusion of autophagosome and lysosome.

**Figure 6:**
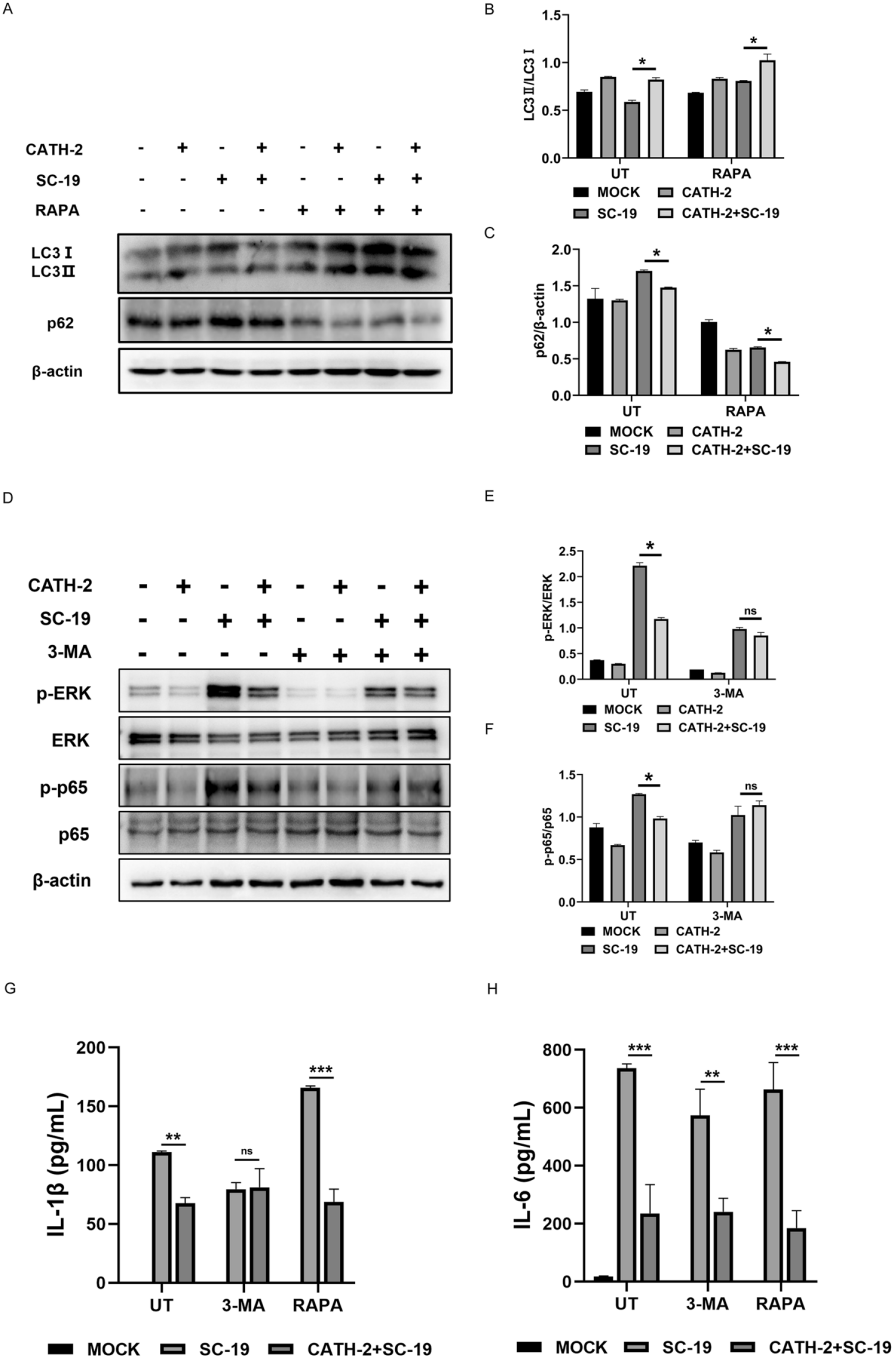
**CATH-2 inhibits SC-19-induced inflammatory response through mTOR-mediated autophagy in macrophages.** Cells were pre-incubated with RAPA (100 nM) for 1 h prior to CATH-2 (1.25 μM) treatment followed by SC-19 infection at an MOI of 5 for 1 h. Western blotting was performed to detect the expression of proteins, including LC3 and p62 (**A**). ImageJ was used to quantify the LC3-II/LC3-I (**B**) and p62/β-actin (**C**) ratios. Cells were pre-incubated with 3-MA (5 mM) for 1 h prior to CATH-2 (2.5 μM) treatment followed by SC-19 infection at an MOI of 5 for 1 h. Western blotting was performed to detect the expression of proteins, including p-ERK, ERK, p-p65, and p65 (**D**). ImageJ was used to quantify the p-ERK/ERK (**E**) and p-p65/p65 (**F**) ratios. Cells were pre-incubated with 3-MA (5 mM) or RAPA (100 nM) for 1 h prior to CATH-2 (2.5 μM) treatment followed by SC-19 infection at an MOI of 5 for 6 h. Then, gentamicin (250 μg/mL) was added to kill extracellular bacteria for 18 h. After 24 h of infection, cell supernatants were collected. ELISA was performed to determine the level of IL-1β (**G**) and IL-6 (**H**). Data presented as mean ± SEM of three independent experiments with triplicate samples per group. *P ≤ 0.05, **P ≤ 0.01, ***P ≤ 0.001, ns = not significant.

It has been reported that autophagy controls inflammation via degradation of critical inflammatory signaling molecules. We further investigated whether CATH-2 inhibits SC-19-induced inflammatory response via autophagy activation. RAPA and 3-MA were used to activate and inhibit autophagy, respectively. The results showed that RAPA promoted CATH-2-induced LC3 upregulation and enhanced CATH-2-induced p62 downregulation during SC-19 infection (Figures 6A–C), demonstrating that CATH-2 induces mTOR-dependent autophagy. Notably, autophagy inhibitor 3-MA diminished the inhibitory effect of CATH-2 on SC-19-induced expression of p-p65 and p-ERK (Figures 6D–F). Similarly, 3-MA blocked the inhibitory effect of CATH-2 on IL-1β secretion while RAPA promoted its inhibitory effect on IL-1β secretion during SC-19 infection (Figure 6G). However, 3-MA and RAPA did not affect the inhibitory effect of CATH-2 on IL-6 secretion (Figure 6H). These results indicate that CATH-2 inhibits the NLRP3/MAPK/NF-κB signaling pathway by regulating mTOR-mediated autophagy.

## Discussion

**Figure 7:**
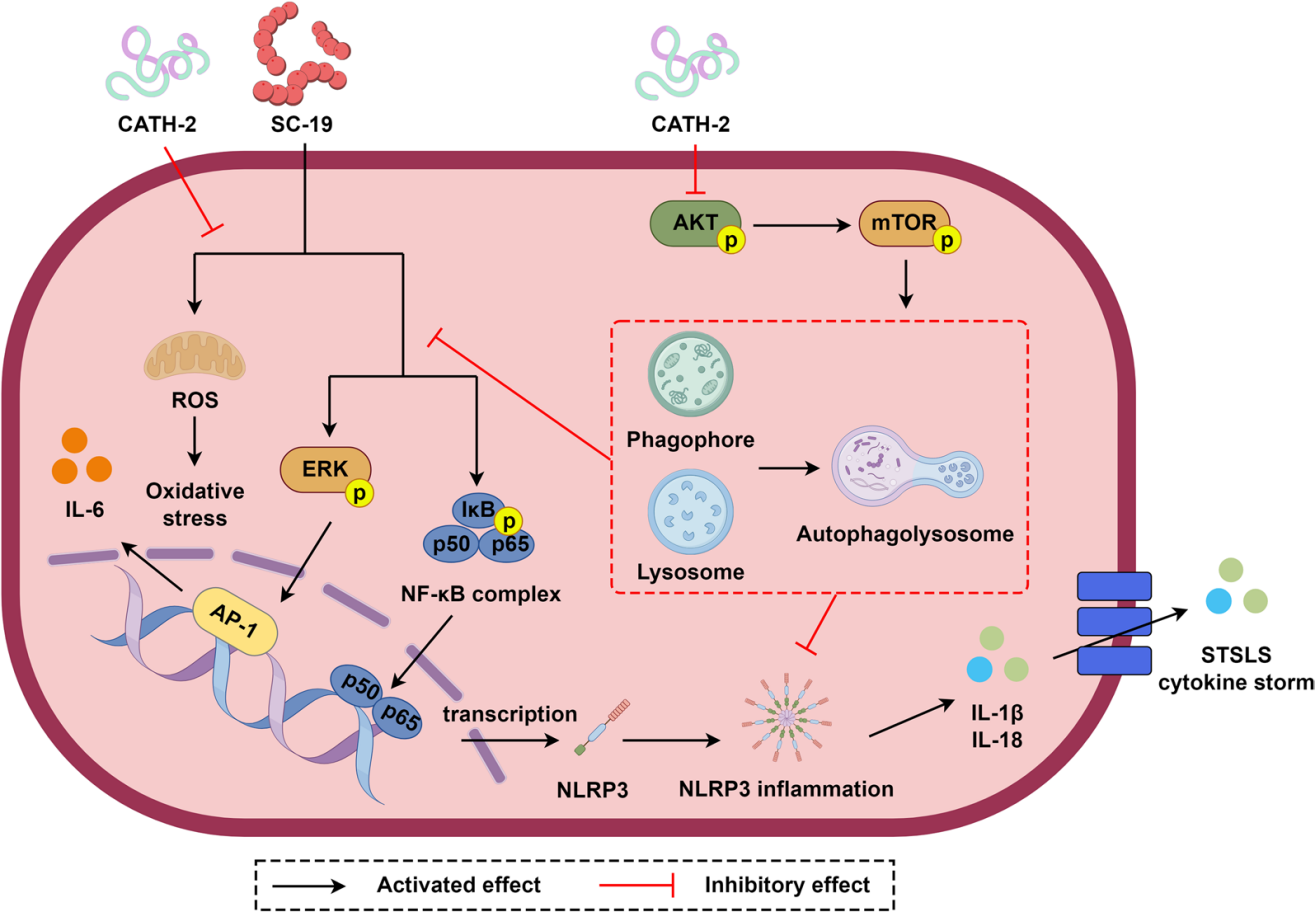
**A working model by which antimicrobial peptide CATH-2 suppresses** ***Streptococcus suis***-**induced inflammatory response via regulating mTOR-dependent autophagy**. *S. suis* infection activates NF-κB/MAPK/NLRP3 and produces ROS, resulting in inflammatory response. CATH-2 pretreatment inhibits phosphorylation of AKT and mTOR to activate autophagy, resulting in the reduction on *S. suis*-induced NF-κB/MAPK/NLRP3 activation and ROS production, thereby inhibiting production of inflammatory cytokines.

Although AMPs are present in different species, their relative structural conservation means that they have similar functions (Figure 7). The formation of the α-helix structure in AMPs is closely related to their interaction with target cell membranes. For instance, in plant AMP snakins [35, 36], plant defensins [37], and animal AMPs bovine myeloid antimicrobial peptide-27 (BMAP-27) [38] and cathelicidin-related antimicrobial peptide (CRAMP) [39], the α-helix is associated with robust membrane disruption. CATH-2 has an α-helical structure and exhibits a broad spectrum of antimicrobial activity [28]. In addition, the anti-inflammatory activity of CATH-2 constitutes a key component of its anti-infective functions, synergizing with direct antimicrobial action to enhance host defense. CATH-2 has been reported to exert its anti-inflammatory effects by neutralizing LPS and LTA, which is refined as a “silent killing” effect on *Pseudomonas aeruginosa* to prevent lung inflammation [40]. Similarly, direct exposure of CATH-2 and bacteria leads to inhibition of *S. suis*-induced activation of murine bone marrow-derived macrophages and dendritic cells [27]. However, the precise mechanism underlying the anti-inflammatory effect of CATH-2 by modulating the host immune response instead of direct interaction with bacteria remains unclear. To investigate the direct immunomodulatory effect of CATH-2 on the host and avoid the direct interaction between CATH-2 and bacteria, cells were pretreated with CATH-2 followed by *S. suis* infection in the current experimental setup. Our study demonstrated that CATH-2 suppressed the NF-κB/ROS/NLRP3-mediated inflammatory response through mTOR-dependent autophagy during *S. suis* infection.

Activation of the NLRP3 inflammasome induces inflammatory and pyroptotic cell death. It has been reported that *S. suis* activates the NLRP3 inflammasome and results in an inflammatory cytokine storm, which leads to STSLS [26], demonstrating that NLRP3 is an important target in the process of bacterial pathogenesis. Therefore, pharmacological inhibition of NLRP3 activation is a promising therapeutic approach to control hyperinflammation-mediated tissue damage. Our study found that CATH-2 reduced *S. suis*-induced NLRP3 activation, which is similar to our previous study on the inhibitory effect of CATH-2 on *E. coli*-induced NLRP3 activation [41]. NLRP3 inflammasome activation firstly requires NLRP3 transcription induced by the engagement of pattern recognition receptors (PRRs) that activate NF-κB, and then recruitment of ASC and caspase-1 occurs in the primed cells to fully activate the inflammasome [42]. Our study showed that CATH-2 inhibited *S. suis*-induced NF-κB activation while CATH-2 also reduced ACS oligomerization, suggesting a dual inhibitory effect of CATH-2 on NLRP3 priming and assembly. This inhibitory effect is similar to that of other AMPs such as frog-derived Brevinin-1FL and snake-derived cathelicidin-WA that exert anti-inflammatory function via inhibiting NF-κB and the NLRP3 inflammasome [43, 44]. Meanwhile, our previous study showed that CATH-2 promotes NLRP3 inflammasome activation in LPS-primed cells [19], which is similar to the property of human cathelicidin LL-37 that has been reported to drive NLRP3 activation in LPS- or *Pseudomonas aeruginosa*-primed cells [45, 46]. These results indicate that CATH-2 exerts a dual role with anti-inflammatory and pro-inflammatory effects under different stimulation conditions.

It has been reported that ROS production induces NLRP3 inflammasome activation. Yu et al. [47] have shown that excessive mitochondrial ROS (mtROS) production in macrophages activates the NF-κB pathway and induces pro-inflammatory differentiation of macrophages. To maintain redox homeostasis, the host eliminates ROS through antioxidant mechanisms by which CAT catalyzes the decomposition of H_2_O_2_ and SOD1 inactivates superoxide and hydrogen peroxide [48]. Our study showed that CATH-2 reduced *S. suis*-induced ROS production by upregulating oxidative scavenging genes including *CAT* and *SOD1*. The inhibition of ROS resulted in reduced IL-1β secretion, demonstrating that ROS mediate the inflammatory response. Mice cathelicidin CRAMP has been reported to alleviate acute ulcerative colitis by increasing the antioxidant enzyme glutathione peroxidase (GSH-PX) to reduce oxidative stress [49]. Cathelicidin has also been reported to alleviate hyperoxia-induced lung injury by inhibiting oxidative stress, which upregulates SOD1 protein expression and downregulates IL-6 secretion [50]. These findings reveal that ROS induce inflammatory injury and cathelicidin reverses oxidative stress by different antioxidant mechanisms to attenuate inflammatory injury.

In addition to antioxidant mechanisms through antioxidant enzymes and oxidative scavenging molecules, mitophagy or autophagy is considered to be an important regulator of inflammatory response, as it removes damaged and dysfunctional mitochondria as well as inflammatory molecules, thereby reducing mtROS and inflammation [51, 52]. We found that CATH-2 mediated the PI3K/AKT/mTOR pathway from RNA-seq analysis, which has been shown to regulate autophagy and the inflammatory response [53, 54]. van Dijk et al. have reported that CATH-2 upregulates the autophagy pathway in human macrophages using RNA-seq [24]. Our study showed that CATH-2 activated mTOR-dependent autophagy and induced autophagic flux, which is similar to the property of LL-37 on the induction of autophagy. LL-37 induces autophagy to accelerate the phagocytosis and clearance of pathogenic bacteria [55]. During the process of autophagy induction, LL-37 is ubiquitinated and recognized by p62, resulting in LL-37 degradation [56]. Although we observed that CATH-2 induced mTOR-dependent autophagy, this phenomenon primarily relied on pharmacological tools, while genetic methods need to be performed in future study. Simultaneously, the exact types of autophagy, such as mitophagy, and whether CATH-2 interacts with the autophagic pathway need to be further studied.

It is well known that autophagy controls inflammation by removing components of pathogens and cellular damage, or by removing key proteins of the inflammatory pathway [57]. For example, Yang et al. found that sulforaphane attenuates inflammatory liver injury in autoimmune hepatitis mice by promoting autophagic flux [58]. Jiang et al. [59] found that the VANGL2 protein attenuated inflammatory injury in the colon by interacting with NLRP3 and promoting autophagic degradation of NLRP3 by increasing K27-linked polyubiquitination at NLRP3 through recruitment of the E3 ligase MARCH8 in a colitis mouse model. Notably, our study for the first time suggests that CATH-2 inhibited the inflammatory response through autophagy induction. However, whether CATH-2-induced autophagy results in ubiquitination and degradation of inflammatory molecules needs to be further studied. Interestingly, GO analysis showed that CATH-2 mediated cellular process regulation and kinase binding, suggesting that CATH-2 might regulate protein posttranslational modification to modulate inflammatory response, but the exact action needs to be further studied.

It should be noted that this study has not yet systematically verified the critical functional domains of CATH-2 responsible for anti-inflammatory effects through the design of scrambled and truncated peptides. Previous studies have reported that substitution of tyrosine with phenylalanine in CATH-2 completely abolished its LPS-neutralizing activity [60], but functional structural domains of CATH-2 associated with autophagy need to be further studied.

In conclusion, *S. suis* infection activates NF-κB/MAPK/NLRP3 and produces ROS, resulting in inflammatory response. CATH-2 pretreatment inhibits phosphorylation of AKT and mTOR to activate autophagy, resulting in a reduction of *S. suis*-induced NF-κB/MAPK/NLRP3 activation and ROS production, thereby inhibiting production of inflammatory cytokines.

## Supplementary Information

**Additional file 1: Original western blots.**

## Data Availability

All data are available within the article and from the corresponding author on reasonable request.

## Declarations

### Sequence information

The raw RNA-seq data have been deposited in the NCBI Sequence Read Archive (SRA) database [61] under sequence accession no. PRJNA1246244.
