# Supplementary material for: Cathelicidin CATH-2 suppresses the NF-κB/ROS/NLRP3 signaling pathway via regulating mTOR-dependent autophagy during Streptococcus suis infection
Source: Vet Res. 2026 Jan 23;57:32. doi: 10.1186/s13567-025-01694-7 (PMC12911090; doi:10.1186/s13567-025-01694-7)

Figure 2

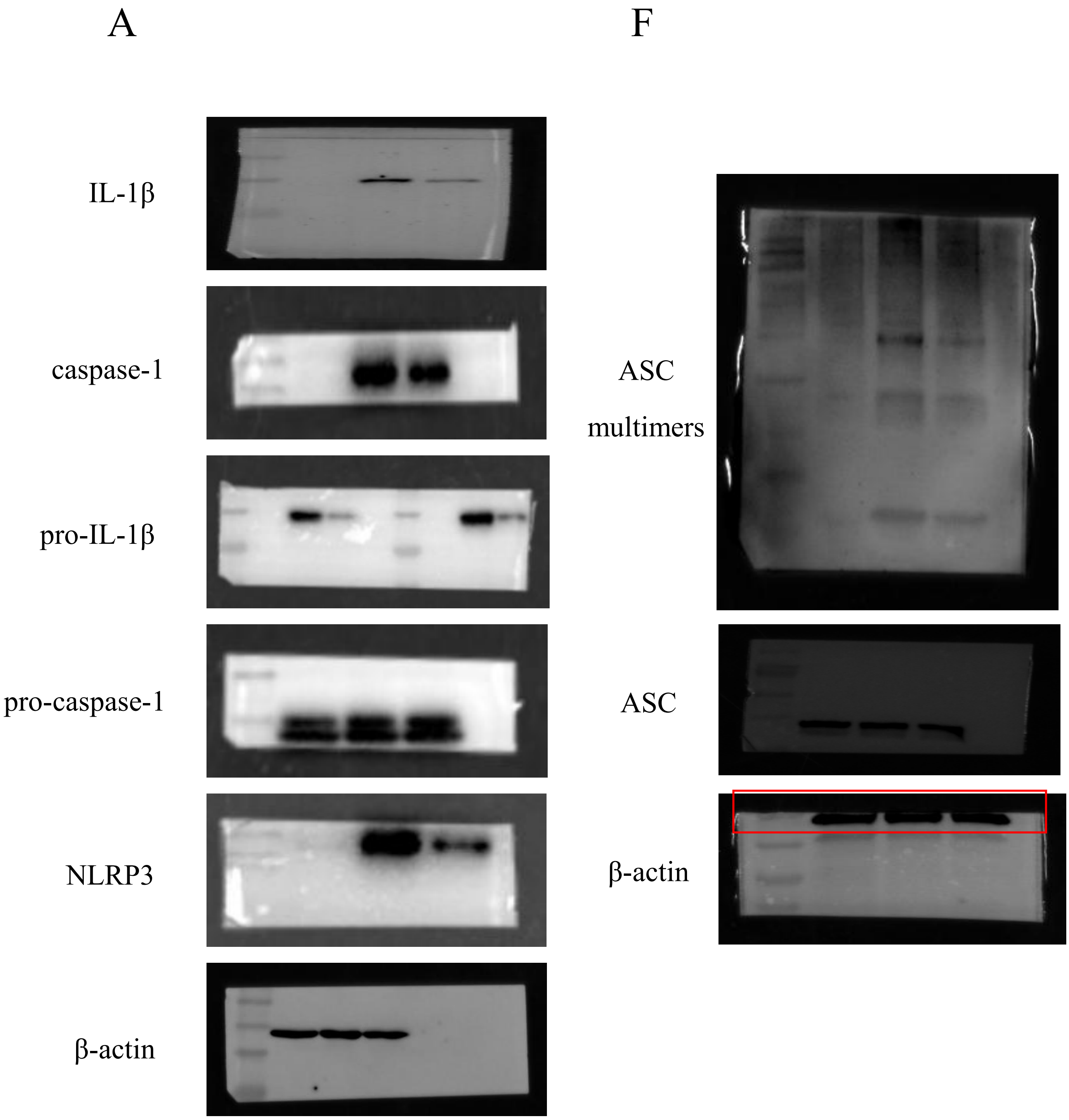
A
IL-1β
caspase-1
pro-IL-1β
pro-caspase-1
NLRP3
β-actin
F
ASC
multimers
ASC
β-actin

Figure 2

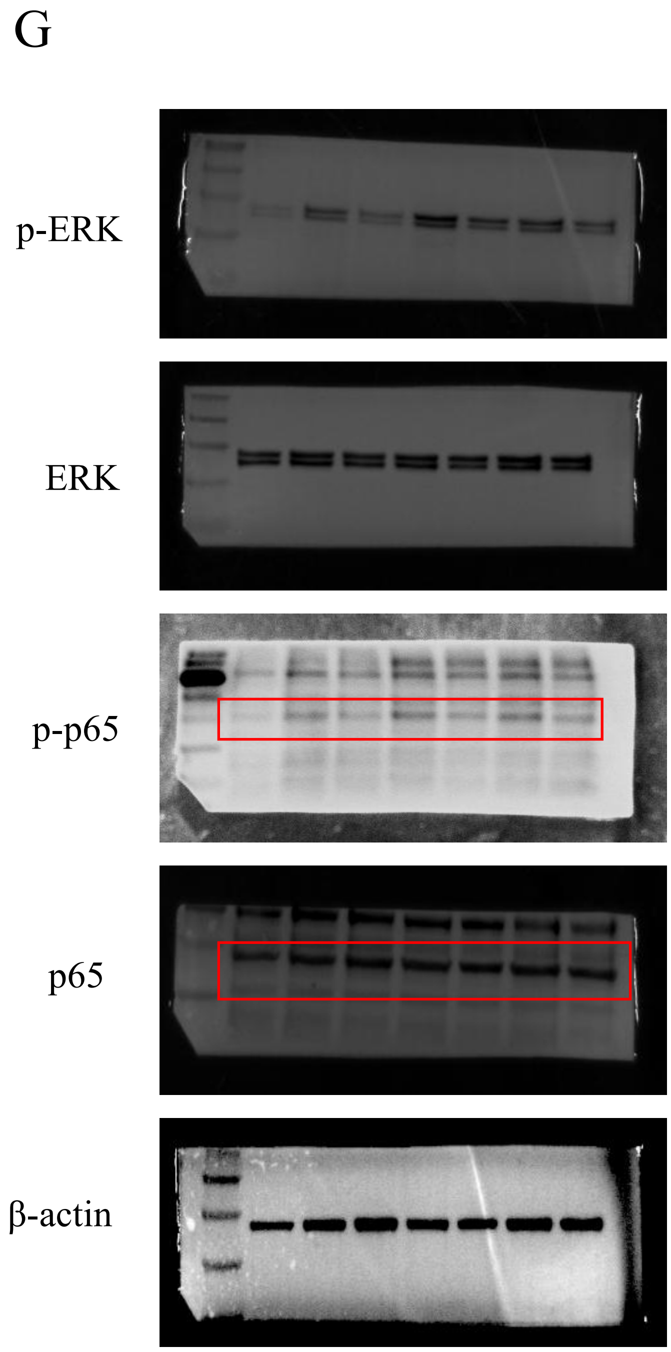

Figure 4

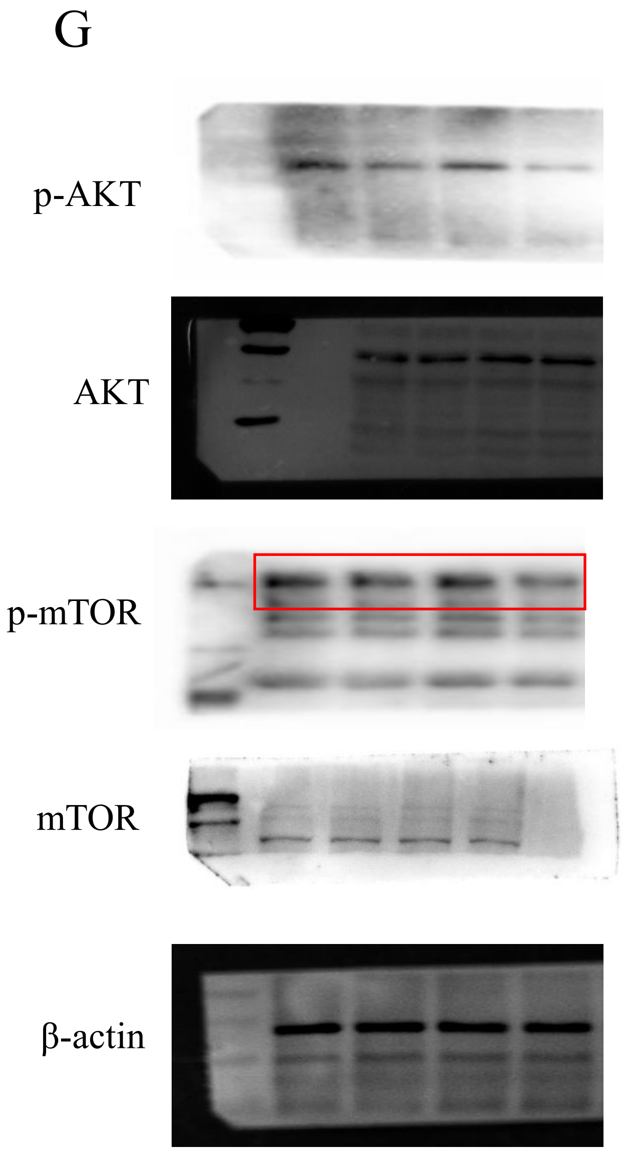

Figure 5

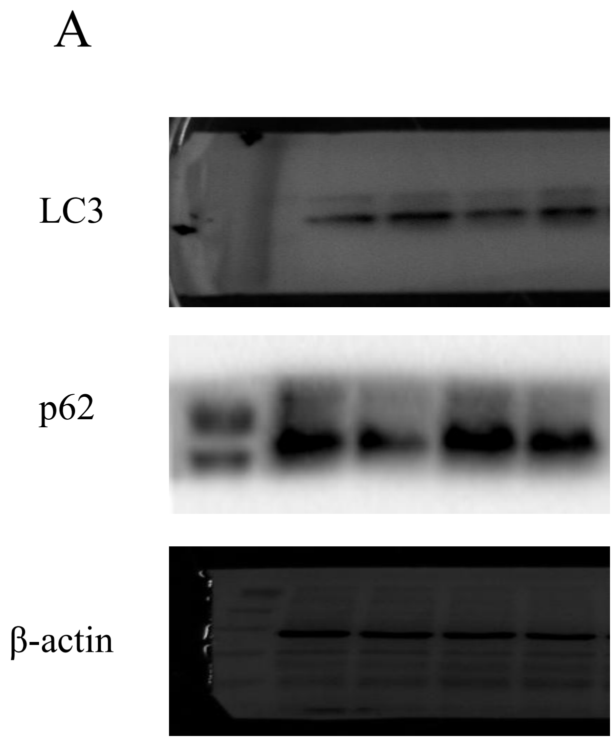

Figure 6

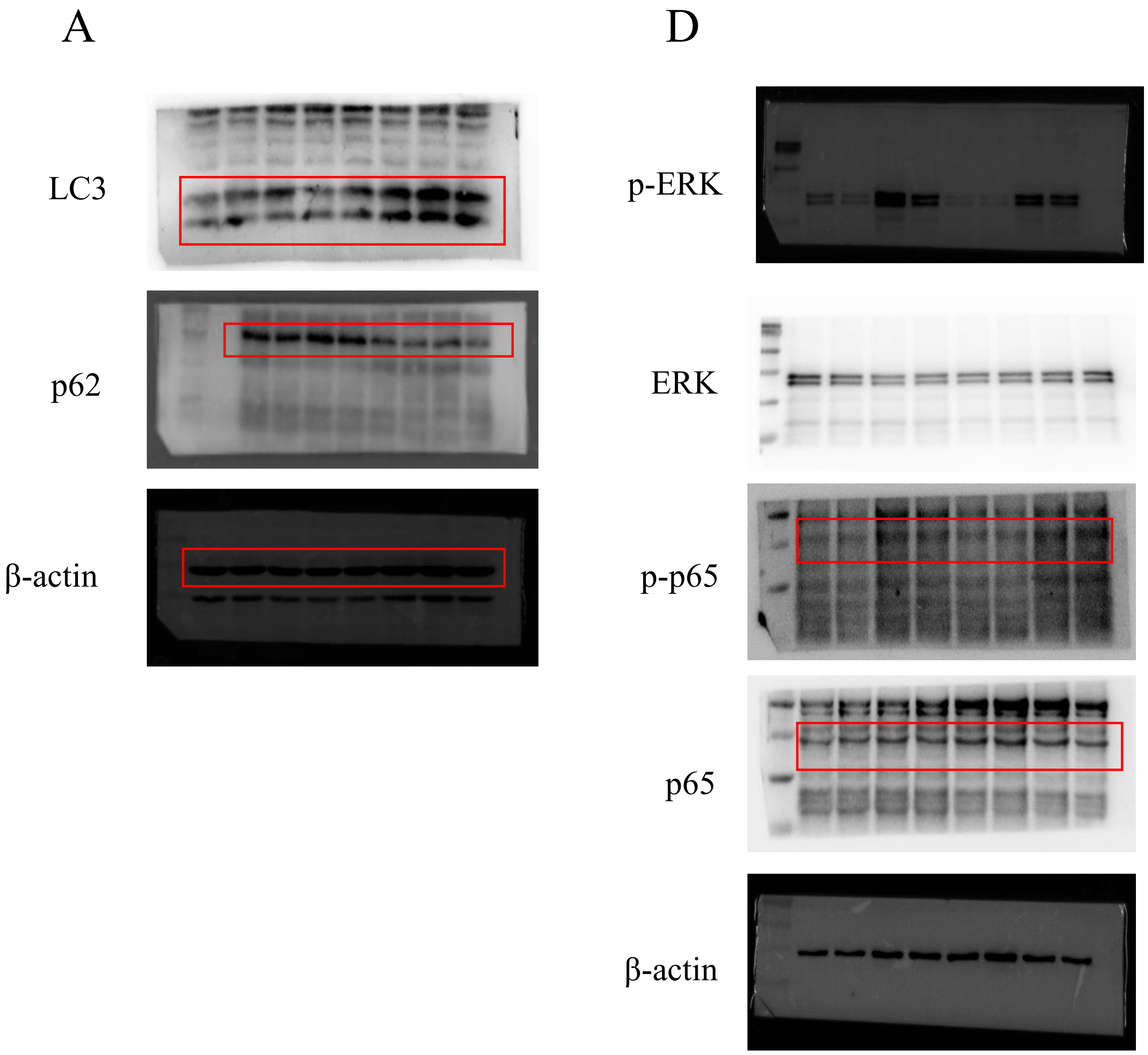

Supplement: Supplementary file 1 — Additional file 1: Original western blots. [file 13567_2025_1694_MOESM1_ESM.pdf]
